# Contributions to Proleptics

**Published:** 1844-07

**Authors:** 


### ART. XIV.

1. *Contributions to Proleptics.* By THOMAS LAYCOCK, M.D., Physician to the York Dispensary, &c. (*Lancet*, vols. i-ii, 1842-3.)

2. *Untersuchungen über periodische Vorgänge im gesunden und kranken Organismus des Menschen.* Von GEORG SCHWEIG.

*Inquiries into the Periodic Occurrences in the Healthy and Morbid Organism of Man.* By GEORGE SCHWEIG.—*Karlsruhe*, 1843. With Five lithographed Tables. 8vo, pp. 166.

3. *Instructions pour l'Observation des Phénomènes périodiques.* Par M. QUETELET, &c. &c. (*Bulletin de l'Académie Royale de Bruxelles*, No. i, tom. ix, 1842.)

*Instructions for the Observation of Periodic Phenomena.* By M. QUETELET.

4. *Instructions pour l'Observation des Phénomènes périodiques de l'Homme.* Par M. SCHWANN, Correspondant de l'Académie Royale de Bruxelles. (*Bulletin de l'Académie*, &c. No. vii, tom. ix.)

*Instructions for the Observation of the Periodic Phenomena of Man.* By M. SCHWANN. (*Bulletins of the Royal Academy of Brussels*, Nos. i and vii, vol. ix.)

THE principal characteristics of the age in which we live are the discovery and development of new truths and the revival of old. The humoral pathology is coming again into vogue, with magnetizing wizards, gothic architecture, railways, steam machinery, illuminated prayer-books, decorated domestic architecture, ceremonial worship, and the conversion of substances into each other, and, of course, of the base metals into gold. Parr's pills are the elixir of life, Liebig, Schultz, and Co. are Rosycrucians, and the Pope hopes to be lord of Christendom once more. In Dr. Laycock's 'Contributions to Proleptics,' (that the movement of the age may be complete,) we have an avowed attempt to re-establish the ancient practice of predicting events, fossilized, to use his words, in the quackery of judicial astrologers, nativity-casters, &c.; not by means, however, of the mysterious agencies of occult science, but simply by a philosophical examination of the order in which phenomena arise and succeed each other. Dr. Laycock would have the law of sequence so investigated that we should be able to anticipate events, national and individual, just as we anticipate day and night, the storms of the equinoxes, the rising and setting of the sun, moon, and stars, the ebb and flow of the tide. He wishes proleptics to be understood as the art and science of predicting, deriving the term from the compound Greek verb prolambano, *anticipo*, I seize beforehand, anticipate. In the contributions before us he endeavours to develop the laws of recurrence of phenomena, drawing his facts from astronomy, meteorology, natural history, physiology, and pathology. We shall trace his views rather according to the order of his periods than of his "contributions."

*The first period or "basic unit."* This comprises a lunar or barometric day of twelve hours, being marked by one maximum and one minimum variation of the barometer. The subjoined table constitutes a condensed view of some of the general facts from which the definition of this period is estimated; we would premise, however, that the proofs of each are given in detail.

"Table of the Meteorological and Physiological Events occurring at the Barometric Hours, during a Solar Day of Twenty-four Hours.

*Four to five o'clock a.m.*

Barometer at its minimum height.
Minimum of electric tension, nearly.
Intermediate minimum variation east of magnetic needle.
Minimum of temperature.
Hour at which several genera of flowers bloom.
Hour when certain species of moth escape from the chrysalis.
Minimum consumption of oxygen.
Outset of cholera, epidemic diarrhea, egyptian ophthalmia, and quotidian ague.
Period of increased excitement of the insane commences.
Hour of alleviation of symptoms and of sleep in hectic and infantile fever.

*Four to five o'clock p.m.*

Barometer at its minimum height.
Minimum of electric tension.
Minimum variation east of magnetic needle.
Certain moths escape from the chrysalis.
Termination of a paroxysm of quotidian ague.
Exacerbation of fevers, accession of hectic fever.
Period of increased excitement in the insane begins.

*Eight to ten o'clock a.m.*

Barometer at its maximum height.
Maximum of electric tension.
Maximum variation east of magnetic needle.
Maximum excitability of the circulation.
Maximum of muscular power.
Period of increased excitement in the insane ends.

*Eight to ten o'clock p.m.*

Barometer at its maximum height.
Maximum of electric tension.
Maximum variation east of magnetic needle.
Meteoric lightning and thunder storms appear.
Certain insects escape from the chrysalis.
Consumption of oxygen at its minimum.
Minimum of muscular power.
Minimum excitability of the circulation.
Hour of natural sleep.
Period of increased excitement in the insane ends.
Paroxysm of a quartan ends." (Lancet, vol. i, 1842-3. p. 931.)

The period thus defined constitutes the primary or basic unit; doubled as in the preceding table, it forms the solar day or quotidian period; quadrupled, the tertian period, &c.

*The period of seven days.* This is made up of two weeks of lunar days, a lunar week constituting the quartan period. This hebdomadal or heptal cycle, according to Dr. Laycock's views, governs, either in its multiple or submultiple, an immense number of phenomena in animal life. The phases of development in insects appear to present the most uniform examples of its influence. In these Dr. Laycock makes four principal periods; 1, the hatching of the ova; 2, the caterpillar or larva state, and the moults which take place at that stage of development; 3, the pupa or chrysalis period; 4, the imago state or puberty.

The ova are hatched in periods varying considerably as to length. The shortest is a lunar week, or three days and a half, as in the wasp, common bee, and ichneumon; in the *cecidomia tritici*, it is two lunar weeks, in the black caterpillar and gooseberry grub, *tenthredo capræa*, three lunar weeks. The larva state rarely occupies less than two or more than twenty-four lunar weeks, and the moults of that state have usually an interval of two lunar weeks.

"The period spent in the pupa state is the most in accordance with the general law of limitation by weeks; in fact, the more exact the observations are as to the length of this period, the more confirmatory are they of the general rule: for example, Mr. Denny had three larvæ of the *sphinx atropos*, which went into the earth on August 22d, 24th, and September 2d, respectively. They appeared as perfect moths on October 16th, 18th, and 27th; or in each case, in exactly eight weeks." (Lancet, vol. i, 1842-3, p. 125.)

The sexual functions of the adult insect (the imago state) exhibit the agency of the same law. Thus twenty or twenty-one days after the queen bee has begun to lay the eggs of drones, the bees begin to construct royal cells. If her impregnation be retarded beyond the twenty-first (Huber), or the twenty-eighth day (Kirby and Spence), of her whole existence, she lays male eggs only, showing then no jealousy of the young queens. Some insects attain puberty immediately after leaving the puparium; others occupy a definite number of weeks in growing, especially the coleopteræ, arachnida, and crustacea. Thus, the newly-disclosed imago of the *cetonia aurata* remains a fortnight under the earth; and that of the *lucanus cervus* not less than three weeks. The common cyclops is at first nearly spherical, and provided with no more than two antennæ, and four short feet. On the 14th day, a small projection appears on the hinder part of the body; on the 22d it acquires a third pair of extremities; and on the 28th it moults.

The periods of incubation or of development of the ova of fishes have not been closely observed, with the single exception of those of the salmon. From Dr. Knox's industrious zeal, we learn that the ova of that fish are hatched in exactly twenty weeks, or 140 days. These periods in birds are much better known to us; they are all regulated by this "heptal" law. The eggs of small birds, as fly-catchers, sparrows, &c., are hatched in two weeks; of gallinaceous birds,—the common fowl, pheasant, grouse, &c.—in three weeks; of the duck tribe in four weeks, of swans in six weeks.

The incubation of the ovum of mammals (the period of utero-gestation) continues also a definite number of weeks. Dr. Laycock has published a list of 129 species of birds and mammals, which proves (as he maintains) the operation of the general law, remembering how difficult it is to calculate the period of utero-gestation to a day.* It is not, however, the phases of development only that exhibit the law of periodicity just laid down; there are various other changes and functions amenable to it. The moults of adult insects, as the arachnida, myriapoda, and crustacea, the exuviation of serpents, and the renewal of the

* Treatise on the Nervous Diseases of Women, p. 48, seq.

dermoid appendages in birds and mammals, are all regulated by it more or less. And so also are minor processes. The ring-pigeon not only sits fourteen days, but lays eggs previously to sitting for fourteen days. Birds of the goose and duck kind lay eggs in the wild state at tertian intervals, that is to say, seven in fourteen days, or one every other day. The goldfinch builds its nest in three days, and it is left unoccupied for four, the first egg not being laid until the 7th day from the beginning.

From the preceding statements, our readers will be able to infer the extensive class of phenomena to which Dr. Laycock applies his law. Dr. Laycock insists that man, in his reproduction and development, is no exception to this law. He looks upon menstruation as analogous to the heat of mammals, and the period of oviposition in birds; but maintains that although in the human female the intervals between each oviposition is four weeks, the " basic unit" is one week. It is certain that in many, the period is three weeks, in others five weeks, while some have a white and red discharge alternately every two weeks. The whole period of utero-gestation (incubation) is ten menstrual periods, or forty weeks.

The chain of demonstration thus carried up link by link, would be manifestly incomplete if the periods of disease did not correspond with, and corroborate those of health. Aware of this, Dr. Laycock proceeds to examine those periodic phenomena observed by physicians from time immemorial, during the course of fevers, both remittent and intermittent. Dr. Laycock takes up the consideration of fevers generally; he shows that the periods of exanthematous fevers are for the most part heptal, that the 4th, 7th, 14th, 17th are critical days in smallpox; that the exanthematous typhus is a twenty-one day fever, that shingles run their course in fourteen days, and that pemphigus, rubeola, scarlatina, &c., are all amenable to the general law.

Of course no one will deny the periodicity of intermittents; Dr. Laycock argues that the remittent and malignant fevers are identical in origin with the former, and therefore subject to the same general law.

"It has been found all over the world that intermittent, remittent, and malignant fevers pass one into another. In Spain and in India, in the West Indies, and in Sicily, in Africa, and in America, this fact has been substantiated by such numerous and competent observers as to set the question at rest. If the febrific poison be concentrated, and the temperature high, the fever is intense in a few hours or days, often indeed without a remission. But if the malaria be less concentrated, and the temperature comparatively low, as in Spain for example, at the beginning of summer and the end of autumn; or if the locality be an elevated one within the tropics, then the fever will be less malignant; probably a regular remittent or an intermittent. . . . . In temperate latitudes the remissions of a malignant remittent, become more complete towards the end of summer, until at last we have quotidian agues; later on, these assume the tertian type; and far into autumn, quartans take the place of tertians." (Lancet, vol. i, 1842-3, p. 126.)

Dr. Laycock compares the order of the critical days of fevers, with the paroxysms of intermittents, as in the following table:

| | |
|---|---|
| "The critical days according to Hippocrates are | 1 . 4 . 7 . 9 . 11 . 14 . 17 . 20 or 21. |
| "The days on which paroxyms of a tertian will take place are | 1 . 3 . 5 . 7 . 9 . 11 . 13 . 15 . 17 . 19 . 21. |
| " Ditto of a quartan . . . | 1 . 4 . 7 . 10 . 13 . 16 . 19. |

"Supposing a remittent fever has the quartan type, and that after the fourth paroxysm an intermission takes place, on what day would the amendment appear? Clearly on the eleventh; and the change of symptoms on that day would be judicatory of a mild paroxysm on the thirteenth, and final amendment on the fourteenth. If the intermission occurred after the fifth paroxysm, then the fourteenth would be the critical day; if at the sixth, the seventeenth, if at the seventh, the fever ends on the twentieth day. .... Whatever type a fever may exhibit, there will be a paroxysm on the seventh day, and consequently this day should be distinguished by an unusual fatality or number of crises. For analogous reasons, the fourteenth will be remarkable as a day of amendment." . . . . (Ibid.)

To strengthen his case still more, by proving that the critical days are not confined to malarious or exanthematous fevers, Dr. Laycock analyses the order in which the paroxysms recur in a fit of the gout, and shows it to have a tertian type, terminating in fourteen days, and after seven paroxysms. However, just as a tertian ague may change into a quotidian or quartan or any other type, so may the gouty paroxysm. Dr. Laycock has seen it as a septiman. Indiscretion in diet or treatment may also double or treble the periods, or render them irregular, just as with agues.

Dr. Laycock quotes other pathological phenomena as illustrating the law. A case of tertian and quartan lunacy communicated to him by Dr. Thurnam, of the York Retreat, shows very strikingly the involution of the minor into the major periods, and the influence of the law on the nervous system; other neuroses, as epilepsy, neuralgia, &c., are referred to in detail. The period of incubation of febrific poisons is never more than a lunar month—a curious fact, which Dr. Laycock explains by the law in question. He adopts the hypothesis of a regular sequence of critical days in health continually going on in the system, and predisposing to the outbreak of disease. This sequence begins with conception. In the embryo it is marked by the successive stages of development in the teeth; in infantile and pubescent life by the successive stages of dentition. If therefore two persons were conceived in the same hour, and developed under the same circumstances, as might happen in the case of twins, the periods of the two would be coincident, and the phenomena of health and disease would be exhibited in both at the same time. An interesting example of this kind in twin-brothers, answering in all respects to the theoretical details above given, is quoted. We observed lately that a writer on mesmerism in the 'Dublin University Magazine,' noticing instances of this kind, attributes the singular coincidences observed to animal magnetism. The case of the twin-brothers Laustand, sick-nurses in an hospital at Bourdeaux, is one of those referred to. These two were always ill at the same time, and became affected with cataract together. Dr. Laycock explains on the same theory the curious coincidences of disease and death in members of the same family, children by the same mother. The time of conception may so far correspond in each, that the sequences of their periods may have a relation to each other, because they will have a common relation to those of the mother; these latter being defined by menstruation, or the escape of ova from the ovaria.

*The causes of vital periods.* According to Dr. Laycock's views, there may be three classes of periods, arranged according to their causes. The first he terms esoteric, from εσω, intus, within; these depend upon the law of sequence just referred to, and belong essentially to the individual, or are grafted upon the organism by the operation of exoteric or external periodic agencies. When the latter cease to act, the sequence of periods still goes on in virtue of "the habit of recurrence" of vital phenomena displayed by animals. The innate and congenital sequence is the most recondite, and as the number seven has so striking a part in the measurement of them, Dr. Laycock refers specially to the ancient doctrine of the harmony of numbers, and to astrology. The operation of the number seven is singularly prominent in all the literature, both of the ancient and modern Asiatics. What was really meant by the harmony of numbers and the perfection of the number seven ? Pythagoras defined harmony to be the relation or *just proportion* of all parts, or *the natural order of all things.* Numbers or arithmetic formed the basis of all his science, and by these he measured the proportion and established the natural order of all things. He was a true inductive philosopher, as is proved by the remains of the sciences he introduced into Europe. The works of Boëtius, the last of the Roman writers, contain the Pythagorean philosophy of arithmetic, geometry, and music. The latter is considered altogether on mathematical principles. Sound from instruments can only be produced by the percussion of the air, and there can be no percussion without motion. Music is divided into three kinds, mundane, human, and instrumental. The first is caused by the swift motion of the machine of heaven in the firmament, for it is no proof that the planets do not give out sounds as they move, that we do not hear them,* &c. The second is seen in the mixing or union of the incorporeal vivacity of reason with body. The laws of the third kind Pythagoras discovered by experiments. He investigated the tension of sounds, as brought out by hammers of different weights, or from musical strings of varying length and thickness, and deduced from his investigations the numerical laws of chords. It is probably to the second kind of music that arithmetic had its physiological application. Then and up to a recent period, arithmetic had its prime and composite numbers, its perfect, abundant and deficient, its odd and even, its polygonal, prismatic, and oblong numbers. The perfect numbers of which Dr. Harris says, there are but ten between a unit and a billion, were formed thus : If the geometrical progression 1, 2, 4, 8, &c. be carried on until the sum be a prime number, and that sum be multiplied by the last term of the series, the product is a perfect number. Thus $1 + 2 = 3$, a prime, and $2 \times 3 = 6$, a perfect number. The number 3 was used principally in mythology and astrology, and has more curious properties than any other. The next perfect number was

* This magnificent philosophical hypothesis, that the planets, by their motions, must strike the ethereal medium around them, and so produce sounds, and that the sounds must be musical, because the motions vary in force and swiftness according to fixed laws, has been often used as a beautiful poetic idea, without any knowledge or at least acknowledgment of its author:

"Sit, Jessica : look how the floor of heaven
Is thick inlaid with patines of bright gold;
There's not the smallest orb, which thou beholdst
But in his motion, like an angel, sings,
Still quiring to the young-eyed cherubims:
Such harmony is in immortal souls;
But whilst this muddy vesture of decay
Doth grossly close it in, we cannot hear it." *Merchant of Venice.*

that used so extensively in physiology and pathology. $1 + 2 + 4 = 7$, a prime number, and $7 \times 4 = 28$, a perfect number; for its aliquot parts are $1 + 2 + 4 + 7 + 14 = 28$. Now let us refer to Dr. Laycock's summary of his minor periods. $1 =$ the day of twelve hours; $2 =$ the solar or quotidian period; $4 =$ the tertian period; $1 + 2 + 4 = 7$, a lunar week and the quartan period.

To take the critical days for another comparison.

Let $s =$ the solar day, or quotidian period,
$2s =$ the tertian,
$4s =$ the quartan, and $1s + 2s + 4s =$ the 7th day or solar week, constituting the prime of a perfect number, and $7s \times 4s = 28s$, the menstrual period. Or to put the series of periods into the Pythagorean series of a perfect number,

$1s + 2s + 4s + 7s + 14s = 28s$, the menstrual period.

We might add illustrations of this kind; the above, however, will show the true bearing of the doctrine of numbers applied to vital acts. Physiology and pathology were manifestly brought under the same numerical laws as the other sciences more purely mathematical. There is harmony of numbers in all nature; in the force of gravity, in the planetary movements, in the laws of heat, light, electricity, and chemical affinity, in the forms of animals and plants, in the perceptions of the mind. The direction indeed of modern natural and physical science is towards a generalization which shall express the fundamental laws of all, by one simple numerical ratio. We would refer to Professor Whewell's 'Philosophy of the Inductive Sciences,' quoted by Dr. Laycock, (vol. i, p. 427,) but particularly to Mr. Hay's researches into the laws of harmonious colouring and form. From these it appears that the number seven is distinguished in the laws regulating the harmonious perception of forms, colours, and sounds, and probably of taste also, if we could analyse our sensations of this kind with mathematical accuracy. We think modern science will soon show that the mysticism of Pythagoras was mystical only to the unlettered, and that it was a system of philosophy founded on the then existing mathematics, which latter seem to have comprised more of the philosophy of numbers than our present.

So far as regards the causes of esoteric periods; the exoteric causes are principally meteorological. We have already noticed those which are diurnal; in investigating the cause of weekly and monthly periods, Dr. Laycock considers the question of lunar influence. He first gives a historical sketch of the doctrines held by the ancients and moderns, and collects numerous facts bearing directly or indirectly on the subject. The general result of his inquiries seems to favour the idea that the moon has an indirect influence on vital acts. The lunar periods had evidently some influence in originating the doctrine of septenaries. From the earliest ages they were used to measure time, as by the Babylonians and Chaldeans. The week of seven days was adopted by all nations who derived their knowledge from these. The Tartar tribes, including the Chinese, the ancient Mexicans, &c. adopted a week of five days. Mr. Cullimore explains some of the characters on the Babylonian bricks, by assuming the latter to be calendars. The characters on these bricks are seven in number, and were originally part of the symbols of the ten primary elements. The other three were the hieroglyphics of the triune deity; the first light, represented by a horn, or a single arrow-head, or a winged egg; the second represented wisdom; the third, love, intelligence, or imparted wisdom. The seven remaining symbols used for the calendar were those of the sun, moon, and five planets, in the order of the days of the week. It appears from a passage in Isaiah, that at Babylon there were monthly prognosticators—*ex-professo;* and Mr. Cullimore thinks that many of these bricks contained the meteorological predictions of this class of men. The latter had reference probably to the moon's influence. Those who are curious in matters of this kind will find more information on the subject in Dr. Laycock's papers than in any work we know of.

The transition from the meteorological influence of the moon to that of the sun is easy. We subjoin the following table exhibiting the influence of the seasons:

| | *Maximum.* | *Minimum.* |
|---|---|---|
| Variations in the barometer: | | |
| *a.* Near London, 1807 to 1816 . | December. | July. |
| *b.* In the Deccan . . | December or January. | July. |
| Variation in the hygrometer . . | January. | July. |
| ,, thermometer . . | July or August. | January. |
| Amount of evaporation . . . | July. | January. |
| Number of births (Belgium) . . | February. | July. |
| ,, deaths (Belgium) . . | January. | July. |
| Cases of Insanity . . . | June or July. | December or January. |
| ,, of Suicide . . . . | Summer. | Winter. |
| Crimes against persons . . | June. | January. |
| ,, property . . | December. | July. |

We also extract the following passage as being worthy attention:

"Correct estimates of the seasonal influence in each year become of much higher importance, when it is remembered that *it is from data of this kind the difficult problem of the periodic return of epidemics must be solved.* The difference between the *medical* constitution of each year is as great as the difference in the meteorological constitution, demonstrated by Mr. Howard .It becomes necessary, therefore, to obtain accurate statistical data of each year for a series of years, (at least for eighteen,) so constructed that the epidemical constitution may be compared with the meteorological." (Lancet, vol. ii, 1842-3, p. 829.)

Dr. Laycock discusses the special physiological and pathological changes induced by the seasons, but maintains that as there is a "habit of recurrence" in daily periods, so there is in annual periods; and quotes examples of the exact punctuality of migrant animals, fishes, birds, and mammals, in proof.

In discussing the septenary periods, Dr. Laycock first gives the literary history, then the actually existing knowledge we have. He reviews the whole life of man, but particularly that portion between birth and middle age. The critical days of infants, the first and second dentition, the evolution of the sexual organs, and the outbreak of hereditary disease at certain periods, are all considered in detail. The literary history displays the same use of the number seven as we have noticed in discussing the minor periods.

*Periodic cycles.* Under this head we have a dissertation on periods of years. The subject is full of interest to the physiologist, naturalist, and even statesman.

"Periods of years may be determined in various ways. The science of astronomy, as we have just seen, marks them; and so also do changes in the electricity and magnetism of the earth and air. Thus, comets, earthquakes, meteors, violent thunder-storms, hurricanes, immense floods, thick fogs, great heat and cold, &c., may be instrumental in determining these periods. The latter phenomena may react upon animated nature, and induce famine, fatal and wide-spreading epidemics, the sudden migration, or extraordinary multiplication, or mortality of animals, as locusts, mice, &c. Famine and pestilence may react upon society at large, and lead to civil strife, revolutions, wars, and the migrations of nations." (Lancet, vol. ii, 1842-3, p. 430.)

The periods, according to Dr. Laycock, may possibly be astrological; on this point a curious fact is stated to the effect, that the violent storm in October last was predicted astrologically for some time previously.

Mr. Howard has discovered a lunar cycle of seventeen or eighteen years by a digest of the barometric observations made daily at Somerset House. He found that the barometric mean is depressed on an average of years by the moon's position in south declination. This depression is gradual, and commences with the moon in full north declination. The effects of this influence are seen through a period of eighteen years. The mean weight of the atmosphere increases through the fore part of this period, and, having kept for a year at the maximum thus obtained, decreases through the remaining years to a minimum, about which there is some fluctuation before the mean begins to rise again. The revolution of the lunar nodes and apogee takes place within this cycle of eighteen years, and Toaldo thought the half of it, or nine years, would explain the recurrence of plagues, &c. The Rev. W. B. Clarke, having collated a large number of historical facts bearing on the subject, came to the certain conclusion that there is a positive periodicity in the derangement of the earth and atmosphere. So far as regards the measure of the periods of recurrence, his views resemble those of Mr. Howard; but he includes the phenomena of earthquakes, famine, pestilence, extraordinary irruptions of animals, &c. in his law. It is rather a curious coincidence that the interval between two epochs distinguished for the phenomena just mentioned, namely 1348-9 and 1833, is filled by exactly twenty-seven of Mr. Howard's lunar cycles. Indeed, there are ample reasons why this period should be recognized at once as established.

Dr. Laycock traces the history of cycles of years in like manner as he has traced the history of smaller periods. He shows that the ancients carried out this branch of proleptics to a great extent. They adopted an extremely general and hypothetical law of periodicity, by virtue of which all mundane phenomena were included within one great cycle or annus magnus of upwards of 300,000 years. At the end of this period everything (they said) in this world began over again. The whole assemblage of celestial phenomena, which are regarded as the influential causes of all changes in the sublunary world, being restored to the same initial order, and proceeding in the same catenation as before, the whole series of events that depend upon them follow in their former connexion of place and time. The same individuals are doomed to be born again, the same cities builded and destroyed, the same arts invented. If this doctrine be true, we have been manifestly in the wrong in denying the claim of a notorious individual to originality on this very point. No doubt the law is his, and was discovered by him above 300,000 years ago. But a sad fatality will attend him and us, for as many years hence he will be at his present follies, and we shall be crowning him with foolscap:

"Alter erit tunc Tiphys ............
.............. erunt etiam altera bella."

II. Schweig's researches are intended to illustrate the periodic movements exhibited in the excretion of uric acid, in the uterine functions, in the number of deaths from various diseases, and in the recurrence of epileptic attacks.

The body of an individual is continually undergoing a change in its composition, new particles being deposited as the old are taken up. The chemistry of this change has been investigated by recent experimenters in various modes; and we have presented to our readers the general results of these inquiries. We refer to our review of Schultz's publication as more particularly discussing the relations of these changes to time. Schweig terms the periods within which they occur tropical periods, from the Greek τρεφω, nutrio, and, after discussing the question at length, adopts the quantity of uric acid excreted as the exponent of the intensity of the changes. He then details his investigations and their results. The following is the method he adopted:

"The hour when the urine was passed was exactly noted, the quantity of the latter measured, and its specific gravity ascertained while yet warm. After the mucus has been removed by filtration, fifteen drops, or if the urine was concentrated twenty drops, of English sulphuric acid, previously re-distilled, were added to every fifty grains of urine, and the whole mixed carefully so as not to wet the sides of the glass vessel. The mixture was then covered up, and was allowed to remain for from thirty-six to forty-eight hours in a cool place, then filtered, ten more drops of sulphuric acid added, and the mixture again set aside for twenty-four hours, so that any remaining uric acid might be precipitated. This part of the experiment is in general really unnecessary, but it is better to do it, so that there can be no doubt that all is precipitated. The crystals remaining on the filter must be washed in distilled water, dried, weighed, and the result duly entered in the journal." (pp. 21-2.)

Schweig finds that the first filtration is absolutely necessary to remove the mucus and epithelial cells; it both facilitates the precipitation of the uric acid, and renders the results accurate. The glass of the vessel should be thin and not scratched, for in the latter case, the crystals of uric acid attach themselves to the scratches. Even if a mark be made on the glass, the surface not being roughened, the crystals will collect upon it. These and other practical hints are given.

The first inquiry is for the purpose of discovering the influence of diurnal periods on the excretion of uric acid. Schweig made 1520 observations on his own urinary secretion in the winter of 1841 and 1842. His life was regular; his three meals a-day were between six and seven in the morning, twelve and two, and between six and seven in the evening. The following table gives the result:

| | | | | Gramme. | | | | | Gramme. |
|---|---|---|---|---|---|---|---|---|---|
| "Night | 12 to | 1 | o'clock | —— | Morning | 6 to | 7 | o'clock | 0·0147 |
| ,, | 1 | 2 | ,, | —— | ,, | 7 | 8 | ,, | 0·0183 |
| ,, | 2 | 3 | ,, | —— | ,, | 8 | 9 | ,, | 0·0170 |
| ,, | 3 | 4 | ,, | 0·0080 | ,, | 9 | 10 | ,, | 0·0136 |
| ,, | 4 | 5 | ,, | 0·0130 | ,, | 10 | 11 | ,, | 0·0120 |
| ,, | 5 | 6 | ,, | 0·0167 | ,, | 11 | 12 | ,, | 0·0110." |

"If the time be considered the ordinate, and the above numbers the abscissa, two curves are formed, a greater and a less. The latter begins at midnight, reaches its maximum between eight and nine in the morning, and ends at noon, where the larger curve begins, reaching its maximum between four and five o'clock p.m., and ending at midnight." (p. 35.)

Each of these curves has a deflection; in the larger it reaches its maximum about six or seven o'clock in the evening, in the smaller about seven in the morning. Schweig refers these to the influence of the rising and setting sun; the quantity decreasing as the sun sets, and increasing at his rising. This table can be compared with Dr. Laycock's. It will be remembered that the observations were made in winter. The average quantity of uric acid excreted per hour was 0·016 gramme. From this, it varied between nothing and 0·053 gramme.

Schweig inquired into the mortality at various hours of the day. The following table shows the result:

*Morning.*

| | 4—5 | 5—6 | 6—7 | 7—8 | 8—9 |
|---|---|---|---|---|---|
| Winter . . | 95 | 109 | 116 | 115 | 113 |
| Summer . . | 120 | 119 | 99 | 100 | 117 |

*Evening.*

| | 4—5 | 5—6 | 6—7 | 7—8 | 8—9 | 9—10 |
|---|---|---|---|---|---|---|
| Winter . . | 103 | 114 | 81 | 77 | 105 | 111 |
| Summer . . | 111 | 119 | 132 | 82 | 86 | 110 |

Comparing these data with the preceding, Schweig says they exhibit an unmistakable similarity.

The following tables show the hour of the day most influential in determining death from consumption:

"*Deaths in Berlin in* 1836 *from Phthisis.*

| | | | |
|---|---|---|---|
| Midnight to 6 o'clock a.m. . . | . 165 | Ratio per 1000 . | . 220 |
| 6 o'clock a.m. to noon . | . 220 | „ . | . 294 |
| Noon to 6 o'clock p.m . | . 197 | „ . | . 263 |
| 6 o'clock p.m. to midnight | . 167 | „ . | . 223 |
| Total | 749 cases | . . | 1000 |

*Deaths from Phthisis in Carlsruhe in* 11 *Years.*

| | | | |
|---|---|---|---|
| Midnight to 6 o'clock a.m. . . | . 187 | Ratio per 1000 . | . 239 |
| 6 o'clock a.m. to noon . | . 218 | „ . | . 280 |
| Noon to 6 o'clock p.m. . | . 195 | „ . | . 250 |
| 6 o'clock p.m to midnight | . 180 | „ . | . 231 |
| Total | 780 cases | . . | 1000." |

The following shows the hourly deaths of the preceding:

| | | | | | | | | |
|---|---|---|---|---|---|---|---|---|
| "Night | 12 to | 1 | . 21 | Noon | 12 to | 1 | . | 42 |
| „ | 1 | 2 | . 27 | „ | 1 | 2 | . | 31 |
| „ | 2 | 3 | . 34 | „ | 2 | 3 | . | 29 |
| „ | 3 | 4 | . 32 | „ | 3 | 4 | . | 27 |
| „ | 4 | 5 | . 43 | „ | 4 | 5 | . | 30 |
| „ | 5 | 6 | . 30 | „ | 5 | 6 | . | 36 |
| Morning | 6 | 7 | . 44 | Evening | 6 | 7 | . | 32 |
| „ | 7 | 8 | . 29 | „ | 7 | 8 | . | 20 |
| „ | | 9 | . 52 | „ | 8 | 9 | . | 22 |
| „ | 9 | 10 | . 37 | „ | 9 | 10 | . | 31 |
| „ | 10 | 11 | . 30 | „ | 10 | 11 | . | 33 |
| „ | 11 | 12 | . 26 | „ | 11 | 12 | . | 42." |

The next table is from Guerry's paper on suicide, in the 'Annales d'Hygiène publique,' Jan. 1831.

"*Hours at which Suicides destroyed themselves.*

| | | | | | | | |
|---|---|---|---|---|---|---|---|
| Midnight | 12 to | 2 | 77 | Noon | 12 to | 2 | 32 |
| ,, | 2 | 4 | 45 | ,, | 2 | 4 | 84 |
| ,, | 4 | 6 | 58 | ,, | 4 | 6 | 104 |
| Morning | 6 | 8 | 135 | Evening | 6 | 8 | 77 |
| ,, | 8 | 10 | 110 | ,, | 8 | 10 | 84 |
| ,, | 10 | 12 | 123 | ,, | 10 | 12 | 71 |
| | | | 548 | | | | 452." |

The minimum is at noon, the maximum from six to eight o'clock a. m. Schweig observes that the influence of diurnal periods in menstruation was first investigated by Metzler. (Versuch einer mediz. Topographie der Stadt Sigmaringen; Freiburg, 1822, p. 365.) At his request, seven unmarried females took note of the day and hour they menstruated. The following is the tabular result:

| | | | | | | | |
|---|---|---|---|---|---|---|---|
| "About | 1 | o'clock a.m., | — times. | About | 1 | o'clock p.m., | 2 times. |
| ,, | 2 | ,, | 3 ,, | ,, | 2 | ,, | 7 ,, |
| ,, | 3 | ,, | 9 ,, | ,, | 3 | ,, | 11 ,, |
| ,, | 4 | ,, | 11 ,, | ,, | 4 | ,, | 6 ,, |
| ,, | 5 | ,, | 4 ,, | ,, | 5 | ,, | 6 ,, |
| ,, | 6 | ,, | 8 ,, | ,, | 6 | ,, | 6 ,, |
| ,, | 7 | ,, | 1 ,, | ,, | 7 | ,, | 2 ,, |
| ,, | 8 | ,, | 1 ,, | ,, | 8 | ,, | 5 ,, |
| ,, | 9 | ,, | — ,, | ,, | 9 | ,, | 3 ,, |
| ,, | 10 | ,, | — ,, | ,, | 10 | ,, | 1 ,, |
| ,, | 11 | ,, | — ,, | ,, | 11 | ,, | — ,, |
| ,, | 12 | ,, | — ,, | ,, | 12 | ,, | — ,, |
| | | Altogether 37 cases, or 44 per cent. | | | | Altogether 49 cases, or 56 per cent." | |

Schweig thus tabulates 246 instances which came to his knowledge.

| | | | | | | | | | |
|---|---|---|---|---|---|---|---|---|---|
| "Night | 12 to | 1 | o'clock, | 1 | Noon | 12 to | 1 | o'clock, | 8 |
| ,, | 1 | 2 | ,, | — | ,, | 1 | 2 | ,, | 13 |
| ,, | 2 | 3 | ,, | 4 | ,, | 2 | 3 | ,, | 14 |
| ,, | 3 | 4 | ,, | 13 | ,, | 3 | 4 | ,, | 19 |
| ,, | 4 | 5 | ,, | 15 | ,, | 4 | 5 | ,, | 14 |
| ,, | 5 | 6 | ,, | 13 | ,, | 5 | 6 | ,, | 14 |
| Morning | 6 | 7 | ,, | 20 | Evening | 6 | 7 | ,, | 7 |
| ,, | 7 | 8 | ,, | 8 | ,, | 7 | 8 | ,, | 8 |
| ,, | 8 | 9 | ,, | 17 | ,, | 8 | 9 | ,, | 13 |
| ,, | 9 | 10 | ,, | 14 | ,, | 9 | 10 | ,, | 9 |
| ,, | 10 | 11 | ,, | 8 | ,, | 10 | 11 | ,, | 4 |
| ,, | 11 | 12 | ,, | 7 | ,, | 11 | 12 | ,, | 3 |
| | | | | 120 | | | | | 126." |

Wetzler connected the maxima and minima with the fluctuations of the barometer: Schweig denies the influence of atmospheric pressure, and shows that menstruation is governed by the same law as the excretion of uric acid, and the hour of dissolution.

Schweig has three major periods, namely, of five days, six days, and seven days. The period of six days is the basis of the other two, and each day is named after the Roman numeration, primus, secundus, tertius, &c. In the five-day period, the third and fourth are made into one day, which he terms *contractus*, and in the seven-day period, a day is interposed between these two, called *interpositus*. The day begins with noon, and not with midnight, for reasons set forth at length. The physiological and pathological value of each of these days are also stated, and with great ingenuity with reference to the excretion of urea, menstruation, fits of epilepsy, the attack of puerperal fever, measles, &c., and the deaths from pneumonia, phthisis, scrofula, &c. Schweig lays down several laws with reference to the pathological conditions mentioned. The urinary secretion exhibits two curves in the cycle of six days, a larger and a smaller. These curves Schweig traces in connexion with the motions of the moon; showing the differences of new and full moon, of apogee and perigee. We extract the following table of 2281 births at the lunar phases, and along with it the statistics of 1403 births in York, by Dr. Laycock; the latter comprise the number happening on three days only at each phase, the day before and after, and the day itself:

| | | |
|---|---|---|
| Hamburgh | New moon to first quarter | 520 |
| | First quarter to full moon | 557 |
| | Full moon to last quarter | 594 |
| | Last quarter to new | 610 |
| York | New moon | 151 |
| | First quarter | 129 |
| | Full moon | 131 |
| | Last quarter | 154 |

The moon's motion is quicker when near perigee than apogee; Schweig found the mortality at Carlsruhe 6 per cent. greater before and after perigee than before and after apogee; the quantity of uric acid secreted is exactly the reverse. From an estimate of numerous facts, Schweig concludes that under certain circumstances, when the intensity of nutrition is at a maximum, the number of deaths is at a minimum, and *vice versâ*.

Some statistical details respecting the menstrual periods are given. The results of 200 menstruations in thirty-four individuals, show an average of 27·8 days, the maximum number in the table being 28 days; in one person in whom menstruation was irregular, sixty-five returns averaged 25·9 days. The relations of menstruation to lunar periods are investigated, and rules deducted; but the instances are too few for any useful purpose of this kind.

The periods of an epileptic are next investigated, the dates of each attack being recorded from January 5th, 1833, to November 7th, 1841. On analysing these, we find a singular complexity of heptal periods, varying in length from 7 days to 56.

In expressing a general opinion of the book under review, we cannot deny its author the credit of great ingenuity and industry. Some of the views are, we think, fanciful, and will not be substantiated; and the complexities of the subject are not sufficiently considered. We think he will derive some valuable information from a perusal of Dr. Laycock's papers.

III. The instructions of M. Quetelet refer, 1, to meteorology and to the physical changes in our globe; 2, to vegetable life; 3, to animal life. Fully sensible of the importance of investigating periodic phenomena on an extended scale, from having himself greatly elucidated them in his ' Essay on Man,' he sought the assistance of philosophic inquirers both at home and abroad. These instructions are published with the intention of securing uniformity in the observations of widely separated observers. M. Quetelet's attention, and that of his collaborateurs, seem to be directed principally to the annual periodic changes; the diurnal ones are expressly neglected. This is done to render the observation less arduous; but we question its policy: it may probably happen that contemporaneous diurnal observations only can render the annual complete. Periodic phenomena have such an infinite variety of relations, that it is impossible even to guess at them *à priori*.

M. Quetelet very wisely dwells on the importance of unity of time and unity of place in making observations, stating that these conditions are indispensable to the deduction of general results.

In looking over M. Quetelet's plan, we were inclined to doubt whether it be sufficiently comprehensive. That part referring to meteorology and vegetable life is more complete than the remaining portion, having reference to animal life. But if we were called upon for a system, we would first recommend a digest of all the observations already made. The various periodical publications, as journals and magazines of natural history, botany, agriculture, and the transactions of learned societies, all contain an immense number of observations already made, which are useless only because isolated. If these were brought together, and tabulated, we are convinced much good would result; not only would general principles be elicited, but we should more distinctly ascertain what ought to be observed. Dr. Laycock, noticing these instructions, suggests several improvements in the third division. The nature of them may be inferred from our analysis of his papers.

The instructions of M. Schwann constitute a supplement to those of M. Quetelet. They refer, firstly, to the periodic phenomena which recur at fixed epochs: these are subdivided into the annual and diurnal, the former including the seasonal influences. M. Schwann would extend the plan of observation to births, deaths, and epidemics, and indeed to all pathological phenomena. The second class of phenomena are those which refer to the epochs in individual life; these include of course, Dr. Laycock's esoperiodic phenomena. M. Schwann would have the organs of the body, brain, lungs, liver, testicles, &c. measured and weighed at all epochs, both during fetal and extra-uterine life. He would also fix the epoch of dentition, puberty, and of involution and decline. It is manifest that in England, M. Schwann's first class of phenomena are all either registered now, or easily may be, by our national system of registration. It only remains with the Registrar-general, as we have shown elsewhere, to connect meteorological phenomena, both diurnal and annual, with movements in the population; the births, epidemics, and deaths from all or from special causes. With regard to the second class, we would observe that M. Schwann's remarks prove that he is not yet acquainted with what has been already ascertained, and consequently, that his "instructions" come with little authority, seeing that he himself wants instructing on some fundamental points.

If the laws of periodicity be considered in all their relations, their mysterious antiquity, their practical importance, their infinite extent, their connexion at once with the most immense phenomena of the universe, and the most minute, they cannot fail to attract philosophic minds to their study. On these grounds, we venture to predict that the art and science of proleptics will rapidly rise into favour. It seems to us, however, an important point that the cultivators of it should have a right bias given them at the beginning. Our Registrar-general, Mr. Chadwick, and the British Association may possibly be brought to co-operate with M. Quetelet and his Belgian friends, and at the next meeting at York, a re-union of those interested in the subject might be so managed as to lead to important practical results. We commend this hint to the parties concerned.

---

